# Multilevel Models for the Analysis of Angle-Specific Torque Curves with Application to Master Athletes

**DOI:** 10.1515/hukin-2015-0105

**Published:** 2015-12-30

**Authors:** Humberto M Carvalho

**Affiliations:** 1 School of Physical Education, University of Campinas, Campinas, Brazil; 2 Faculty of Sport Sciences and Physical Education, University of Coimbra, Coimbra, Portugal

**Keywords:** multilevel regression modeling, isokinetic, muscle function, strength

## Abstract

The aim of this paper was to outline a multilevel modeling approach to fit individual angle-specific torque curves describing concentric knee extension and flexion isokinetic muscular actions in Master athletes. The potential of the analytical approach to examine between individual differences across the angle-specific torque curves was illustrated including between-individuals variation due to gender differences at a higher level. Torques in concentric muscular actions of knee extension and knee extension at 60º·s^−1^ were considered within a range of motion between 5º and 85º (only torques “truly” isokinetic). Multilevel time series models with autoregressive covariance structures with standard multilevel models were superior fits compared with standard multilevel models for repeated measures to fit angle-specific torque curves. Third and fourth order polynomial models were the best fits to describe angle-specific torque curves of isokinetic knee flexion and extension concentric actions, respectively. The fixed exponents allow interpretations for initial acceleration, the angle at peak torque and the decrement of torque after peak torque. Also, the multilevel models were flexible to illustrate the influence of gender differences on the shape of torque throughout the range of motion and in the shape of the curves. The presented multilevel regression models may afford a general framework to examine angle-specific moment curves by isokinetic dynamometry, and add to the understanding mechanisms of strength development, particularly the force-length relationship, both related to performance and injury prevention.

## Introduction

Physiological functions during exercise, generally used to describe populations, compare groups or define changes across the life span, are all conceptualized in respect to time (Rowland, 2011). In particular, the force-length properties of a whole group of synergetic muscles, i.e., a fundamental issue in muscle mechanics, is generally overlooked despite its practical and theoretical relevance (Herzog et al., 1991). The shape of the curves has been mostly described using maximal voluntary muscular action under isometric conditions (Herzog et al., 1991; Philippou et al., 2004, 2012).

Isokinetic dynamometry has emerged as a favoured method both in clinical research and applied sports contexts to provide information about maximal dynamic muscular action when the velocity of the movement is controlled and maintained constant (Gleeson and Mercer, 1996; Tsepis et al., 2004). Maximal moments of force derived from isokinetic dynamometry data appear to be consistently favored as a measure of isokinetic leg strength. However, the importance of interpreting the profile of isokinetic angle-specific moment curves between the injured (such as anterior cruciate ligament) and healthy lower limb is recognized (Tsepis et al., 2004), but seldom reported and limited to case studies. Also, it is important to consider acute shifts in the force-length relationship as consequence of muscle-damaging exercise using angle-specific torque curves and the optimum angle for peak torque generation (Brockett et al., 2001; Brughelli and Cronin, 2007; Philippou et al., 2004; Prasartwuth et al., 2006). Overall, the qualitative and quantitative examination of angle-specific moment curves may be useful in identifying and monitoring patterns of strength development, and potential implications for injury and performance.

Another issue of importance dealing with angle-specific torque curves lies in the nonlinear fitting and the appropriateness of analytical approaches to fit individual and group curves. Both second order (Philippou et al., 2004; Philippou et al., 2009) and fourth order polynomials fitting curves (Prasartwuth et al., 2006) based on repeated measures analysis of variance (ANOVA) and analysis of covariance (ANCOVA) to describe changes in the moments of force across the range of motion have been reported. To the best of our knowledge, no study had examined the impact of different polynomial models fits on the shape of angle-specific torque curves. On the other hand, traditional repeated-measures ANOVA to explore longitudinal physiological data may be limited (Kristensen and Hansen, 2004). The restrictive assumptions of repeated-measures ANOVA may not hold when fitting in angle-specific torque curves, in particular the requirement that correlations among measurements on the same subject satisfy a restrictive condition called sphericity, i.e., equal variability of the measurements at each time point and equal correlations between every two measurements on the same individual (Gueorguieva and Krystal, 2004). Multilevel time series models, based on a standard multilevel model for repeated measures data (Goldstein et al., 1994), may be an alternative approach to fit individual angle-specific moment curves. The multilevel regression models allow the use of realistic yet parsimonious variance and consider the correlation patterns in the repeated measures (Goldstein et al., 1994; Singer and Willett, 2003). In particular dealing with multiple measurements that are made close in time may lead to autocorrelation of within-individuals residuals (Goldstein et al., 1994; Steele, 2008). Thus, the application of multilevel time series models, which is an extension of standard multilevel models for repeated measures including an autocorrelation model for within-individual residuals (at Level-1) (Goldstein et al., 1994), may be suitable to fit angle-specific torque curves. In addition, the approach has the flexibility to allow the consideration of group factors and other covariates to study both how the mean values of the curve parameters change across groups and to what extent group differences and the between-individual variation can be explained by other factors (Goldstein et al., 1994; Singer and Willett, 2003).

The aim of this paper was to outline a multilevel modeling approach to fit individual angle-specific torque curves describing concentric knee extension (KE) and flexion (KF) isokinetic muscular actions in Master athletes. Furthermore, the analytical method was tested to partition the between-individuals variation accounted to gender differences on the angle-specific torque curves.

## Material and Methods

### Sample

The sample included 12 Portuguese Master athletes, 50 to 74 years old engaged in competitive swimming and athletics (female athletes, n = 5, 56.0 ± 3.6 years; male athletes, n = 7, 65.5 ± 5.6 years). The study was approved by the ethics committee of the Faculty of Sport Sciences and Physical Education, the University of Coimbra, and the subjects were informed about the nature of the study, that participation was voluntary and that they could withdraw from the study at any time.

### Isokinetic dynamometry assessment

In the present study torques in reciprocal concentric muscular actions of KF and KE at 60º·s^−1^ from a sample of Master athletes were considered. Isokinetic assessment of reciprocal KE and KF actions were made using a calibrated dynamometer (Biodex System 3, Shirley, NY, USA) at angular velocity of 60º·s^−1^. The warm-up consisted of 10 min cycling on a Monark cycle ergometer (Monark 814E, Varberg, Sweden) with minimal resistance (basket supported) at 60 rev·min^−1^ and 2 min of static stretching of the hamstring and quadriceps muscles. For isokinetic assessment, the athlete was placed in a seated position adjusted according to manufacturer guidelines in standardized 85º hip flexion from the anatomical position. Reciprocal muscular actions in the dominant leg were considered. The lever arm of the dynamometer was aligned with the lateral epicondyle of the knee and the force pad was placed approximately 3 to 5 cm superior to the medial malleolus with the ankle in a plantigrade position. Range of motion during testing was set using voluntary maximal full extension (0º) to 90º of knee flexion. Cushioning was set using hard deceleration (according to manufacturer guidelines) and therefore, 90º constituted the range of motion tested. Effects of gravity on the limb and lever arm were accounted for. Athletes gripped handles at the sides of the Biodex System 3 seat during the test procedure. Each athlete performed one set of five continuous maximal repetitions. Visual feedback of moment versus time was provided during the test, but no verbal feedback was given (Baltzopoulos et al., 1991). KE and KF torque values at each angular position from the repetitions with higher maximal torque were retained for analysis and expressed as N·m. The range of motion considered for analysis was set between 5 and 85º, considering only torques “truly” isokinetic at 60º·s^−1^, and torque was averaged in each angular position (*θ*). All measurements were made by a single experienced observer (Carvalho et al., 2011).

### Statistical analysis

**Figure 1:**
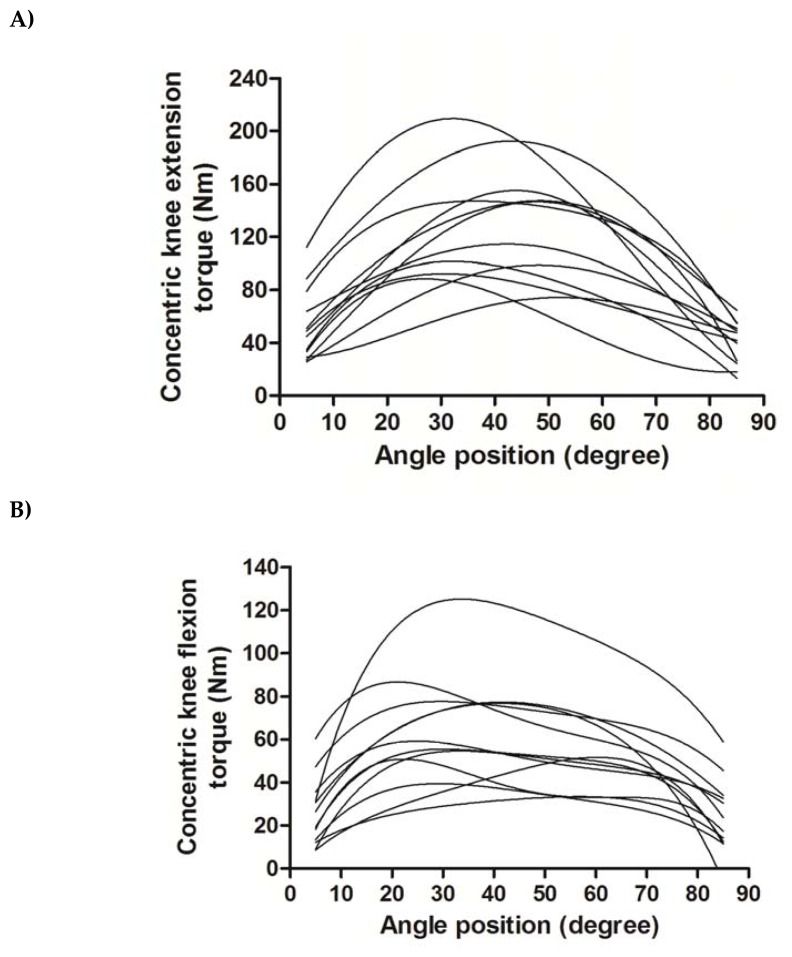
Examining the OLS individual trajectories of isokinetic knee extension (Panel A) and flexion (Panel B) muscular actions at 60º·s^−1^ in master athletes. Note that in Panel A 0º corresponds to knee flexion at 90º as the angle scale was inverted in the models for knee extension curves.

We initially explored individual trajectories trends fitting ordinary least squares (OLS) estimation to individual data (Figure 1). The basic structure of the repeated measures data in this study had a two-level hierarchical structure with measurements (each *θ* within the range of motion) at level 1 nested within individuals at level 2. Thus, the multilevel models described repeated observations over time, in this case over successive *θ* (at level 1) nested within individuals (level 2).

In the second step of the analysis, we explored the appropriateness of standard multilevel regressions against multilevel time series models. Standard multilevel regressions generally assume that all residuals are normally distributed, and that residuals defined at the same level may be correlated. Multilevel time series models are an extension of standard multilevel models for repeated measures that include an autocorrelation model for within-individual residuals (at level 1) (Goldstein et al., 1994; Steele, 2008).

The third step of the analysis compared the unconditional growth model for the second order polynomial (Equation 1) with higher order polynomial models (Equation 2), up to fourth order polynomials. The unconditional growth model for the second order polynomial is described as:

(Equation 1) $$\begin{array}{c} T_{ij}=t_{0i}+t_{1i}(\theta_{ij})+t_{2i}({\theta^{2}}_{ij})+{ɛ}_{ij},\\ t_{0i}=\gamma_{00}+\zeta_{0i}\\ t_{1i}=\gamma_{10}+\zeta_{1i}\\ t_{2i}=\gamma_{20}+\zeta_{2i} \end{array}$$

Denoted by *T_ij_* is the torque at measurement occasion *j* (*j* = 1, ... , *ji*) for individual *i* (*i* = 1, ... , *n*), i.e., each *θ* (angular position) within the observed range of motion. Denoted by *t*_0_*_i_* is the individual *i’*s true initial torque value, i.e., the value when the *θ* is 0. Denoted by *t*_1_*_i_* is the individual *i’*s true linear rate of torque change across the observed range of motion. Denoted by *t*_2_*_i_* is the individual *i’*s true nonlinear rate of torque change (second order) across the observed range of motion. *ɛ_ij_* represents that portion of individual *i’*s outcome that is unpredicted on the *θ_j_* within the observed range of motion. In the level 2 sub-model, *γ*_00_ represents the population average initial torque value; *γ*_10_ and *γ*_20_ represent the linear and nonlinear (second order) rate of torque change within the observed range of motion. The level 2 residuals are denoted by ζ_0_*_i_*, ζ_1_*_i_*, ζ_2_*_i_* that represent deviations of the individual torque change trajectories around their respective group average trend.

The higher order polynomials added to the unconditional growth model are described as:

(Equation 2) $$\begin{array}{c} T_{ij}=t_{0i}+t_{1i}(\theta_{ij})+t_{2i}({\theta^{2}}_{ij})+t_{3i}({\theta^{3}}_{ij})+t_{4i}({\theta^{4}}_{ij})+{ɛ}_{ij},\\ t_{0i}=\gamma_{00}+\zeta_{0i}\\ t_{1i}=\gamma_{10}+\zeta_{1i}\\ t_{2i}=\gamma_{20}+\zeta_{2i} \end{array}$$

The final step of the analysis explored the inclusion of explanatory variables (covariates). As example in this study we examined gender differences, where male athletes were coded 0 and female athletes were coded 1, as well as cross-level interactions (gender × *θ*; gender × *θ*^2^ interaction). The explanatory variables were added as fixed effects to equation 2.

The scale of *θ* for KF curves, i.e. movement starting at a position of full leg extension (0º) until flexion of 90º was set as reference in the isokinetic dynamometry assessment. Thus, for interpretation of KE curves, i.e. movement starting at a 90º position until the full leg extension (0º), the scale of *θ* was reversed to allow interpretation of the initial rate of strength with the *t*_1_ exponent (i.e. represents the linear increase of torque by *θ*) and the *t*_2_ exponent as an indicator of torque decrease after the attainment of the angle of peak torque.

The reference range of motion was considered in the models for the interpretation of KF curves, i.e., movement starting at a position of full extension (0º) until a flexion of 90º. For the KE, i.e. movement starting at a 90º position until the full extension (0º), the scale of *θ* was reversed to allow interpretation of the initial rate of strength with the *t*_1_ exponent (i.e. represents the linear increase of torque by *θ*) and the *t*_2_ exponent as an indicator of the torque decrease after attainment of the angle of peak torque.

Maximum likelihood estimation was used to obtain the parameters. The Akaike’s Information Criterion (AIC) and Bayesian Information Criterion (BIC) were reported and used for model comparison. Additionally, visual inspection of residual plotted to determine the models validity to fit torque values throughout the range of motion. Multilevel regression models were obtained using “nlme” package (Pinheiro and Bates, 2000), available as a package in the R statistical language (http://cran.r-project.org).

## Results

**Figure 2:**
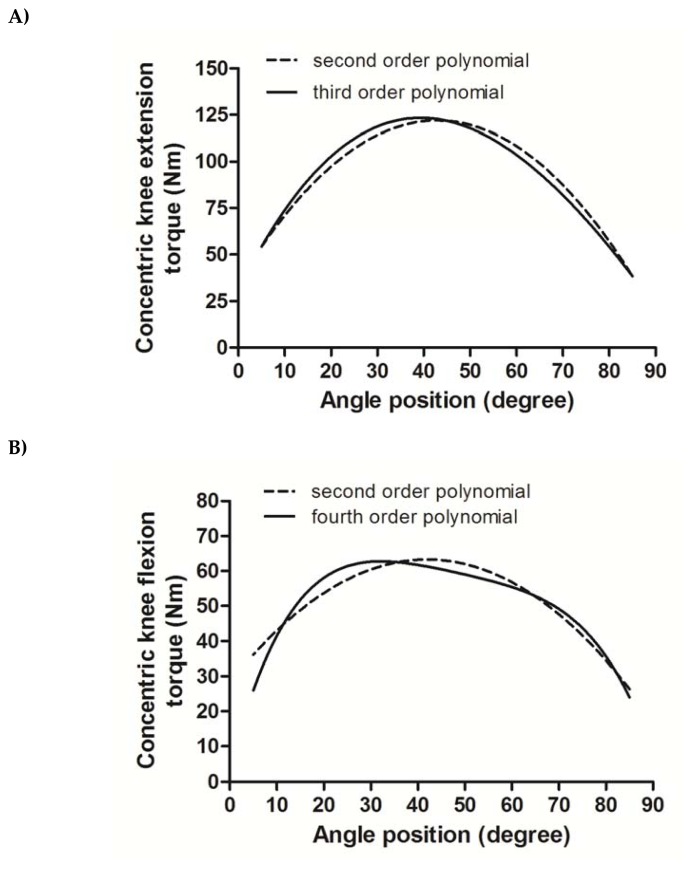
Angle-specific torque curves fitted using multilevel modeling in concentric knee extension and knee flexion isokinetic muscular actions at 60º·s^−1^ in Master athletes (Panel A: predicted concentric knee extension angle-specific torque curves using second order polynomial and third order polynomial fits, note that 0º in Panel A corresponds to knee flexion at 90º as the angle scale was inverted in the models for knee extension curves; Panel B: predicted concentric knee flexion angle-specific torque curves using second order polynomial and fourth order polynomial fits).

The results of model comparison between nonlinear curve fitting with multilevel models to describe KE and KE concentric actions are summarized in Table 1. Smaller value of AIC and BIC for the third degree polynomial model implies that this model was a better fit compared to the second degree polynomial model and fourth degree polynomial model to fit the angle-specific torque curves of isokinetic KE action. As for the isokinetic KF action, the fourth degree polynomial model showed to be the superior fit to describe the angle-specific torque curves. Also, multilevel time series models with autoregressive covariance structures with standard multilevel models had substantially lower AIC and BIC compared with standard multilevel models for repeated measures to fit angle-specific torque curves (multilevel time series model for the best fit KE: AIC = 4103.988, BIC = 4162.565 vs standard multilevel model for comparable KE: AIC = 6348.906, BIC = 6402.601; multilevel time series model for the best fit KF: AIC = 3426.012, BIC = 3489.457 vs standard multilevel model for comparable KF: AIC = 5301.213, BIC = 5359.778). The interpretation of estimates and 95% confidence intervals for initial torque (intercept), the initial rate of torque development (linear θ exponent), attainment of peak torque (second order exponent) indicate that the second order polynomial estimation underestimated at least the initial rate of torque development and tended to overestimate the angle for maximum torque values attainment (Figure 2 and Table 2).

**Figure 3:**
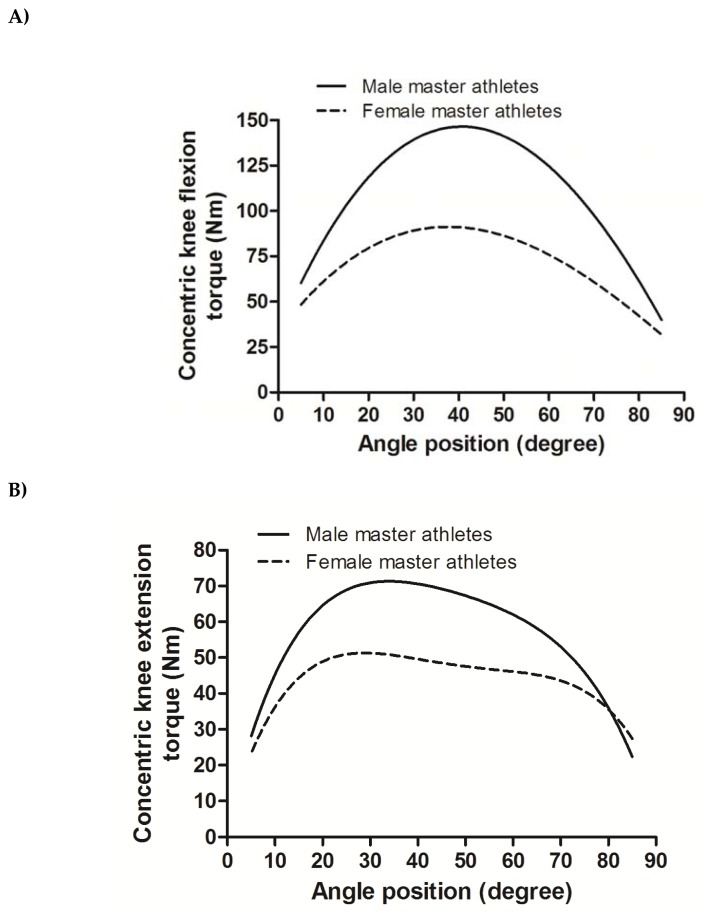
Angle-specific torque curves fitted by gender using multilevel modeling in concentric knee extension and knee flexion isokinetic muscular actions at 60º·s^−1^ in Master athletes (Panel A: predicted concentric knee extension angle- specific torque curve, note that 0º in Panel A corresponds to knee flexion at 90º as the angle scale was inverted in the models for knee extension curves; Panel B: predicted concentric knee flexion angle-specific torque curve).

The inclusion of explanatory variables allows in this example to examine the influence of inter-individual variability of gender on Master athletes’ KE and KF angle-specific strength curves. The equations derived from the multilevel regression model with lower AIC and BIC values are presented in Figure 3 and were as follows:

$$\begin{array}{l} Concentric\;knee\;extension=30.938+6.086\;(\theta )-0.089\;(\theta^{2})+0.0002\;(\theta^{3})-2.466\;(gender\;x\;\theta interaction)+0.028\;(gender\;x\;\theta^{2}\;interaction)\\ Concentric\;knee\;flexion=5.978\;(\theta )-5.771\;(\theta^{2})+0.0002\;(\theta^{3})-0.000011\;(\theta 4)-1.179\;(gender\;x\;\theta interaction)+0.013\;(gender\;x\;\theta^{2}\;interaction) \end{array}$$

All exponents in the equation were significant at p < 0.01. The gender term was not included in the equations since p = 0.56. The intercept term was not included in the equation that described KF, as the term was 2.978 with a standard error of 4.703. The magnitude of the interaction term between gender and the linear term θ exponent indicates that the male athletes had a higher rate of initial moment of strength, i.e. initial acceleration, as well as a higher peak torque value for both KE and KF. Also, the negative exponent interaction term between gender and the θ2 suggests a higher decrement of torque after the attainment of peak torque for male Master athletes.

## Discussion

The present study illustrates the use of multilevel time series models to test higher order polynomial fits of individual angle-specific torque curves on KE and KE isokinetic muscular actions.

Initial ordinary least-squares fits of individual torque-angle data from the Master athletes are shown in Figure 1 for both isokinetic KE (A) and KF (B). Data for KE ranged from 5.4 to 210.0 N·m while flexion data ranged from 126.6 to 0.5 N·m. Models comparison showed that the third order polynomial fit for KE and the fourth order polynomial fit for KF were superior compared to the second order polynomial model fit. Moreover, the inspection of the 95% confidence intervals of the intercept, linear and second order exponents suggests that the models produce different estimates for, at least, the initial value, the initial linear rate of torque and the angle of attained peak torque (Figure 2).

The most frequently used curve fitting procedures of angle-specific torque are the Gaussian fitting curve (Bowers et al., 2004; Brockett et al., 2001), the second order polynomial (Philippou et al., 2004; Philippou et al., 2009; Philippou et al., 2012) and the fourth order polynomial (Prasartwuth et al., 2006). The Gaussian fitting curve requires that data is normally distributed. Usually only data points >75–90% peak torque can be used to produce the curve (Brughelli and Cronin, 2007). Thus, the method is limited to identify the angle of peak torque. The use of polynomial fitting curve allows describing changes across the entire range of motion (Brughelli and Cronin, 2007). The nonlinear procedures used to fit curves have been based on traditional repeated-measures ANOVA (Philippou et al., 2004; Philippou et al., 2009; Philippou et al., 2012; Prasartwuth et al., 2006; Tsepis et al., 2004). The adoption of the statistical approach may be limited to fit isokinetic angle-specific torque curve due to the restrictive assumptions related to the error covariance matrix. In the present study, the results support the use of third and fourth order polynomials to explore the shape of the angle-specific torque curves. It is apparent that the higher order nonlinear fits may be more sensitive to examine the initial rate of force production, peak torque attained and the angle of peak torque, as well as the rate of decline of torque throughout the range of motion after the angle of peak torque. Particularly, the fit of the third order polynomial for the KE was unexpected, suggesting a trend of shift of the angle of peak to the middle of the range of motion. One of the main advantages of this method is the flexibility to model different error covariance structures, particularly in longitudinal data sets where autocorrelation is often present (Goldstein et al., 1994; Singer and Willett, 2003).

To interpret the fixed exponents of KEcon action, it should be noted that range of motion during testing was set using voluntary maximal full extension (0º) starting at 90º of knee flexion. Since the angular position in the model was inverted, the linear angular position term refers to the initial acceleration and the second order angular position term refers to attainment of maximum torque production. The magnitude of the second order exponent may also provide an indication of the ability to maintain the torque production throughout the range of motion after peak torque until the full extension (Figure 1).

The analysis of changes in the mechanical properties of the muscle, particularly the shifts force-length relationship in consequence of acute exercise or training exposure has been of interest to researchers (Brughelli and Cronin, 2007; Osternig, 1986). For example, shifts of the optimum angle for peak torque generation in quadriceps muscles towards longer muscle lengths observed after acute eccentric exercise reflect transient changes in the mechanical properties of this muscle (Philippou et al., 2009). Other relevant applied context of interest to researchers and coaches lies in the influence of age-related changes and long-term exposure to the training stimulus in the neuromuscular characteristics of Master athletes, especially in relevance to the increased importance of athletic performance for older athletes (Baker et al., 2010).

The inclusion of explanatory variables allows in this case to examine the influence of inter-individual variability accounted to gender differences on the shape of isokinetic KE and KF concentric actions. Initially, the inclusion of gender in the models showed a substantial variation between athletes in both muscular actions, where it could be inferred that male Master athletes had higher values of torque across the range of motion compared to female athletes. The inspection of cross-level interactions included in the models exemplifies the flexibility of the multilevel time series models to allow interpreting the possible influence of explanatory variables in the different descriptors of the angle- specific torque curves. The interactions exponents (gender × θ interaction and gender ×θ 2 interaction) suggest that gender differences have at least an influence on the ability to produce higher rates of initial torque and maximal torques. Differences in maximum torque output, the angle of attainment of maximum torque across the range of motion, particularly for the knee extension, have been reported (Pincivero et al., 2004; Suter and Herzog, 1997; Welsch et al., 1998), although data was mainly obtained by fitting curves to isometric muscles actions. Particularly in Master athletes, the use of the multilevel time series model may allow further inspection of the complexities between age-related changes in muscle architecture, age-related contractile properties changes and training exposure.

The analyses in the present study are confined to describe angle-specific torque with an expected shape. Whether it is better to fit a higher polynomial when the shape of angle-specific torque curves deviates from expected, e.g., after anterior cruciate ligament injury, is a matter that should be further investigated.

In summary, we outlined the use of the multilevel time series model to fit individual angle-specific moment curves. The multilevel regression models provide a flexible approach to deal with structures of short time series, allowing the flexibility to explore individual and/or group characteristics at a higher level as explanatory variables. The procedures described here may provide a general framework to examine angle-specific torque curves by isokinetic dynamomentry both for individual and group characteristics, and add to the understanding of the force-length relationship both related to performance and injury prevention.

## Figures and Tables

**Table 1:** Comparison between nonlinear fits using multilevel modeling with autoregressive covariance structures to describe angle-specific moment curves of concentric knee extension and flexion isokinetic muscular actions in Master athletes

*Model*	Degrees of freedom	Akaike’s Information Criterion	Bayesian Information Criterion
Knee extension			
*Model 1*: *T*_ij_ = γ_00_ + γ_10_ (*θ*_ij_) + γ_20_ (*θ* ^2^_ij_) + ζ_0i_ + ζ_1i_ + ζ_2i i_ *+ɛ_ij_*	11	4130.148	4183.843
*Model 2*: Model 1 + *t*_3i_ (*θ* ^3^_ij_)	12	4103.988	4162.565
*Model 3*: Model 2 + *t*_4i_ (*θ* ^4^_ij_)	13	4105.865	4169.323

Knee flexion			
*Model 4*: Y _ij_ = γ_00_ + γ_10_ (*θ*_ij_) + γ_20_ (*θ* ^2^_ij_) + ζ_0i_ + ζ_1i_ + ζ_2i i_ *+ɛ_ij_*	11	3549.111	3602.795
*Model 5*: Model 4 + *t*_3i_ (*θ* ^3^_ij_)	12	3519.852	3578.416
*Model 6*: Model 5 + *t*_4i_ (*θ* ^4^_ij_)	13	3426.012	3489.457

θ represents the angular position

**Table 2:** Comparison between nonlinear fits using multilevel modeling to describe angle-specific moment curves of concentric knee extension and flexion isokinetic muscular actions in Master athletes

	Concentric knee extension	
	
Parameter *(95 % confidence intervals)*	Second order polynomials model	Third order polynomials model
Intercept	35.22 (19.62 to 54.86)	30.91 (11.21 to 50.62)
*θ*	4.03 (3.78 to 4.31)	5.06 (4.61 to 5.51)
*θ*^2^	−0.03 (−0.05 to −0.04)	−0.08 (−0.09 to −0.07)
*θ*^3^	-	0.0002 (0.0001 to 0.0003)
*θ*^4^	-	-

	Concentric knee flexion	
	
	Second order polynomials model	Fourth order polynomials model

Intercept	13.63 (3.57 to 23.70)	8.85 (−1.27 to 19.00)
*θ*	2.33 (2.13 to 2.52	3.70 (3.16 to 4.27)
*θ*^2^	−0.026 (−0.028 to −0.024)	−0.12 (−0.14 to −0.09)
*θ*^3^	-	0.002 (0.001 to 0.002)
*θ*^4^	-	−0.000011 (−0.000013 to −0.000009)

θ represents the angular position
